# Evaluation of *PTPN22* polymorphisms and Vogt-Koyanagi-Harada disease in Japanese patients

**Published:** 2009-06-03

**Authors:** Yukihiro Horie, Nobuyoshi Kitaichi, Yoshihiko Katsuyama, Kazuhiko Yoshida, Toshie Miura, Masao Ota, Yuri Asukata, Hidetoshi Inoko, Nobuhisa Mizuki, Susumu Ishida, Shigeaki Ohno

**Affiliations:** 1 Department of Ophthalmology, Hokkaido University Graduate School of Medicine, Sapporo, Japan; 2 Department of Pharmacy, Shinshu University School of Medicine, Matsumoto, Japan; 3 Department of Legal Medicine, Shinshu University School of Medicine, Matsumoto, Japan; 4 Department of Ophthalmology, Yokohama City University School of Medicine, Yokohama, Japan; 5 Department of Molecular Life Science, Division of Molecular Medical Science and Molecular Medicine, Tokai University School of Medicine, Isehara, Japan; 6 Department of Ocular Inflammation and Immunology, Hokkaido University Graduate School of Medicine, Sapporo, Japan

## Abstract

**Purpose:**

Vogt-Koyanagi-Harada (VKH) disease is an autoimmune disorder against melanocytes. Polymorphisms of the protein tyrosine phosphatase non-receptor 22 gene (*PTPN22*) have recently been reported to be associated with susceptibility to several autoimmune diseases. In this study, genetic susceptibility to VKH disease was investigated by screening for single nucleotide polymorphisms (SNPs) of *PTPN22*.

**Methods:**

A total of 167 Japanese patients with VKH disease and 188 healthy Japanese controls were genotyped by direct sequencing methods for six SNPs (rs3811021, rs1217413, rs1237682, rs3761935, rs3789608, and rs2243471) of *PTPN22* including the uncoding exons.

**Results:**

The six SNPs in *PTPN22* showed no significant association with susceptibility to VKH disease or its ocular, neurologic, or dermatological manifestation.

**Conclusions:**

Further studies are needed to clarify the genetic mechanisms underlying VKH disease.

## Introduction

Vogt-Koyanagi-Harada (VKH) disease is one of the most frequent forms of uveitis in Japan [1]. It is characterized as bilateral panuveitis accompanied by neurologic and skin lesions [2,3]. This disease is considered to be an autoimmune disease against melanocytes [4,5]. Though the etiology of VKH disease still remains unknown, genetic factors may play an important role in susceptibility as indicated by an established association between VKH disease and specific human leukocyte antigen (*HLA*)*-DRB1* alleles [6,7].

The protein tyrosine phosphatase non-receptor 22 gene (*PTPN22*) is located on chromosome 1p13.3-p13.1, and it encodes the lymphoid-specific phosphatase (Lyp) that is important in the negative control of T-cell activation and development [8-10]. Recently, it was reported the single nucleotide polymorphism (SNP), R620W (rs2476601), in *PTPN22* increased susceptibility to several autoimmune diseases including rheumatoid arthritis (RA), systemic lupus erythematosus (SLE), and insulin dependent diabetes mellitus (IDDM) [11-15]. In the *PTPN22* risk variant (rs2476601), this substitution disrupts an interaction between Lyp and the protein tyrosine kinase, Csk, and may translate biologically to a potential for ‘hyperreactive’ pathogenic T-cell responses [8].

This R620W mutation was not observed in the Japanese population [16,17]. Therefore, in this study, we analyzed six SNPs, which belong to the same haplotype block as R620W (rs2476601) in *PTPN22*. For the efficacy of the linkage analysis, we chose six SNPs of which minor allele frequencies were more than 15% from the database of Japanese Single Nucleotide Polymorphisms [18,19].

## Methods

We recruited 167 VKH (72 males and 95 females) patients and 188 healthy controls for this study. All patients and control subjects were Japanese. Patients were diagnosed according to the “Revised Diagnostic Criteria for VKH Disease” [3] at the Uveitis Survey Clinic of the Hokkaido University Hospital (Sapporo, Japan) and Yokohama City University Hospital (Yokohama, Japan). All patients and control subjects were informed of the study’s purpose, and their consent obtained. The study was approved by the ethics committee from each institute participating in this study.

**Figure 1:**
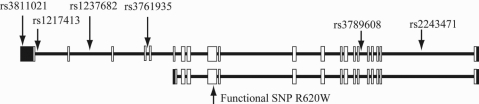
*PTPN22* structure with two transcript isoforms and six SNP. Six SNP variants with minor allele frequencies 15% from the database of Japanese Single Nucleotide Polymorphisms. The black and white areas in the exons indicate the UTR and coding region, respectively.

DNA was prepared from peripheral blood specimens using the QIAamp DNA Blood Mini Kit (Qiagen, Tokyo, Japan). Six SNPs (rs3811021, rs1217413, rs1237682, rs3761935, rs3789608, and rs2243471) from the *PTPN22* region were examined (Figure 1). Each of the six SNPs was amplified by standard polymerase chain reactions (PCRs; Table 1). After purification using ExoSAP-IT (USB Corporation, Cleveland, OH), the PCR products were sequenced with Big Dye Terminator v3.1 (Applied Biosystems, Foster City, CA) using either sense or antisense primers (Table 1). The BigDye XTerminator Purification Kit (Applied Biosystems) was used to purify the DNA from sequencing reactions. The sequencing reactions were analyzed using an ABI3130 sequencer (Applied Biosystems).

**Table 1:** PCR primers for *PTPN22* SNPs.

**SNP**		**position**	**Primers**	**Product size (bp)**	**sequence primer**
rs3811021	(SNP1)	114158186	F: TGGGTTGCAATACAAACTGCTC	600	Forward
			R: TCAATTTGCCCTATTGGACTTC		
rs1217413	(SNP2)	114159273	F: TTGCAGGTGTACTTGCAGCC	552	Forward
			R: TTGAAGGATTTCTGGACCGAC		
rs1237682	(SNP3)	114165627	F: AAGGAGGCACAGATTCCACAC	589	Forward
			R: TGACCATGCCAATATACCAACTG		
rs3761935	(SNP4)	114174051	F: AAAGTTTCCGGCATGTTTCC	595	Reverse
			R: TGGTGATTGTCGGCTAAGATTG		
rs3789608	(SNP5)	114199311	F: CATCATGGTCTGGCCAATTC	589	Forward
			R: TGAGGTGGAGTTCTAACCACAAG		
rs2243471	(SNP6)	114207525	F: GACAAGACTGAATTGTACGAGCG	577	Forward
			R: CACCATCTCCAGCCTCTCAC		

The position of the SNPs is cited from the NCBI database.

### Statistical analysis

For statistical analyses, the Hardy–Weinberg equilibrium was tested for each SNP among the control subjects. Genotype frequency differences between the case and control genotypes were assessed by the χ^2^ test. The calculation of linkage disequilibrium (LD) and pair-wise LD (D’ value) between SNPs of the *PTPN22* region and the haplotypes was performed with Haploview software, version 3.32. The maximum likelihood estimates of haplotype frequencies were estimated by pairs of unphased genotypes using the expectation-maximization (EM) algorithms in the R package ‘haplo.stats’ [20].

## Results

**Figure 2:**
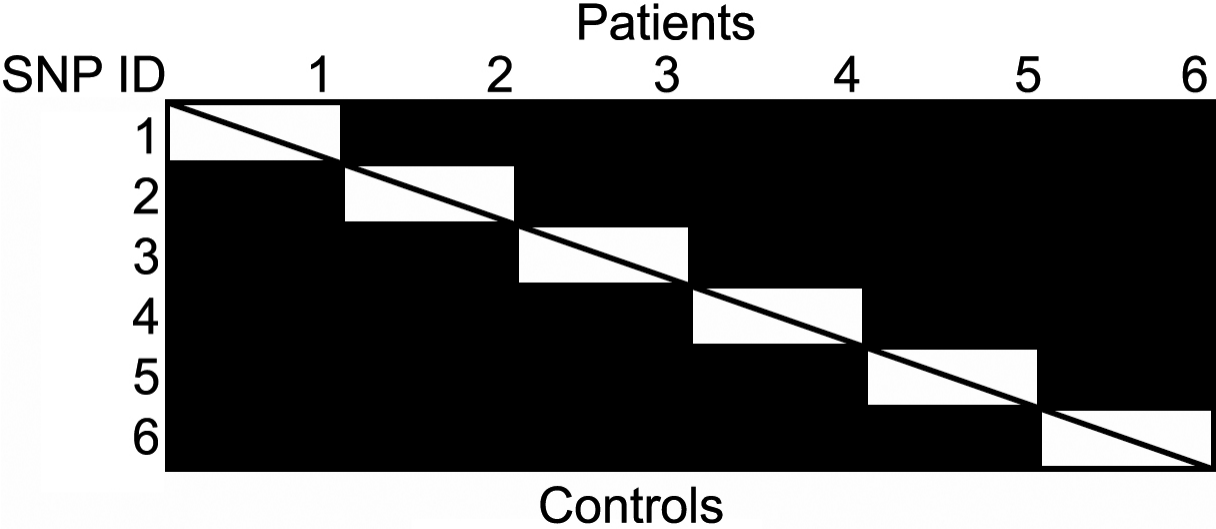
D' score for the six SNPs studied across the *PTPN22* haplotype. Black cells indicate that D' is greater than 0.9. Upper: patient population, lower: control population. The figure indicates that the six SNPs were in all the same haplotype block.

Allele frequencies for the six SNPs covering the gene were in Hardy–Weinberg equilibrium in both the patients and controls. The allelic frequency of each SNP in both groups was nearly equal, and no association was detected when compared independently (odds ratio, OR 1.14–1.35; Table 2). Stratifying the patients by the presence of diffuse choroiditis, sunset glow fundus, nummular chorioretinal depigmented spots, neurologic auditory involvement, meningismus, tinnitus, cerebrospinal fluid pleocytosis, or integumentary findings also revealed no evidence of association in VKH disease (data not shown). We calculated pairwise D’ values for all SNP pairs in *PTPN22* (Figure 2). The pairwise D’ values in the gene were nearly 1 among almost all SNP pairs, indicating the SNPs were highly associated with each other and the entire *PTPN22* was contained within a single LD block. Haplotype analysis predicted and revealed that *PTPN22* was not associated with VKH disease in this Japanese cohort (data not shown).

**Table 2:** Genotype frequencies in VKH patients and controls.

**SNP**	**Allele**	**VKH (n=168)**	**Percentage**	**Control (n=187)**	**Percentage**	**Odds ratio (95% CI)**	**p**
rs3811021	C/C	2	1.2	9	4.8	0.24 (0.05–17.47)	0.05
	T/C	56	33.5	67	35.8	0.90 (0.58–7.38)	0.65
	T/T	109	65.3	111	59.4	1.29 (0.84–7.53)	0.25
	C	60		85		0.75 (0.51–7.41)	0.12
rs1217413	A/A	26	16	36	19.4	0.79 (0.45–7.63)	0.41
	A/G	77	47.2	91	48.9	0.93 (0.61–7.43)	0.75
	G/G	60	36.8	59	31.7	1.25 (0.80–7.56)	0.32
	A	129		163		0.84 (0.62–7.25)	0.26
rs1237682	C/C	26	15.5	33	18.4	0.83 (0.47–7.63)	0.53
	T/C	79	47	89	49.7	0.94 (0.62–7.34)	0.77
	T/T	59	35.1	57	31.8	1.20 (0.77–7.53)	0.42
	C	131		155		0.87 (0.64–7.24)	0.37
rs3761935	G/G	3	1.8	10	5.3	0.33 (0.09–12.76)	0.08
	T/G	55	33.3	67	35.8	0.90 (0.58–7.39)	0.62
	T/T	107	64.8	110	58.8	1.29 (0.84–7.54)	0.25
	G	61		87		0.75 (0.52–7.41)	0.12
rs3789608	T/T	2	1.2	9	4.8	0.24 (0.05–17.57)	0.05
	C/T	57	34.1	68	36.4	0.91 (0.59–7.38)	0.66
	C/C	108	64.7	110	58.8	1.26 (0.82–7.50)	0.29
	T	61		86		0.74 (0.51–7.41)	0.11
rs2243471	A/A	27	16.7	37	20.2	0.79 (0.46–7.62)	0.4
	A/G	79	48.8	88	48.1	1.03 (0.67–7.36)	0.9
	G/G	56	34.6	58	31.7	1.14 (0.73–7.48)	0.57
	A	133		162		0.88 (0.65–7.24)	0.39

The above table is the genotype and allele frequencies of the VKH patients and healthy controls. There are no differences between patients and controls.

## Discussion

In the present study, we analyzed polymorphisms of the new candidate gene, *PTPN22*, in Japanese patients with VKH disease. The gene encodes an important negative regulator of T cell activation [9]. An SNP of *PTPN22*, R620W (rs2476601) was reported to be associated with several autoimmune diseases such as RA, SLE, and IDDM [11,12,14,15]. However, this SNP, which disrupts an interaction between Lyp and the protein tyrosine kinase, Csk, does not exist as a polymorphism in the Japanese population [9,10,12]. Therefore, in this study, we examined six other SNPs to evaluate the susceptibility locus of *PTPN22*. *HLA-DRB1* is a common genetic factor in autoimmune diseases (RA and IDDM). Therefore, there may be other common genetic factors in VKH disease [21].

VKH disease is considered to be an autoimmune disease against melanocytes [2-5]. In early studies, activated T lymphocytes were elevated and attacked melanocytes of ocular choroidal tissue in patients in the active phase of VKH disease [22]. Antigen-specific T-cell assay revealed that peptide fragments of the tyrosinase family proteins (tyrosinase, tyrosinase related protein 1 and 2) proliferated in T lymphocytes collected from VKH patients [4,5]. These proteins are found in human melanocytes. These antigen-specific T cell responses were detected in cells collected from HLA-DRB1*04 positive VKH patients only but not from HLA-DRB1*04 negative patients or HLA-DRB1*04 positive healthy people [7,23]. In the Japanese population, 40% of healthy people have HLA-DRB1*04 [7]. However, people having VKH disease represent only 0.01% of the Japanese population [1,7,24,25]. In addition, some patients with VKH disease are HLA-DRB1*04 negative [7]. Thus, it is believed HLA-DRB1*04 is a major susceptible gene in VKH disease. However, other minor genetic factors still remain unclear. To find other susceptible genes, we studied the tyrosinase gene (*TYR*), tyrosinase related protein 1 gene (*TYRP1*), tyrosinase related protein 2 gene (*TYRP2*), and interferon (*IFN-γ*), but we could not find any association with these genes and VKH disease [7,26]. Genetic influences of VKH were also investigated in other countries, but the etiology of the disease seems to be unresolved [27-29].

In this study, we found no association between *PTPN22* and VKH disease in the individuals studied. Our results suggest that further molecular genetic studies are needed to detect novel genetic loci and predisposing genes and to elucidate the true genetic mechanisms underlying VKH disease.
